# Positron Emission Tomography Imaging Reveals an Importance of Saturable Liver Uptake Transport for the Pharmacokinetics of Metoclopramide

**DOI:** 10.1155/2018/7310146

**Published:** 2018-05-08

**Authors:** Fabien Caillé, Sébastien Goutal, Solène Marie, Sylvain Auvity, Salvatore Cisternino, Bertrand Kuhnast, Géraldine Pottier, Nicolas Tournier

**Affiliations:** ^1^Imagerie Moléculaire in Vivo, IMIV, CEA, Inserm, CNRS, University of Paris-Sud, Université Paris Saclay, CEA-SHFJ, Orsay 91400, France; ^2^Variabilité de Réponse aux Psychotropes, UMR-S 1144, Inserm, Université Paris Descartes, Université Paris Diderot, Paris 75006, France; ^3^Assistance Publique des Hôpitaux de Paris–AP-HP, Paris, France

## Abstract

Positron emission tomography (PET) imaging using [^11^C]metoclopramide, a P-glycoprotein (P-gp) substrate, was used to investigate the contribution of transport processes to metoclopramide liver clearance. The liver kinetics obtained after injection of [^11^C]metoclopramide were measured using PET in rats (*n*=4‐5) in the absence (tracer dose) and the presence of a pharmacologic dose of metoclopramide (3 mg/kg), with or without P-gp inhibition using i.v. tariquidar (8 mg/kg). Corresponding [^11^C]metoclopramide kinetics and metabolism in plasma (*n*=3) were measured using radio-HPLC analysis. [^11^C]metoclopramide exposure to the liver and plasma was described by the area under the time-activity curve (AUC) of the radioactivity kinetics in the liver and parent [^11^C]metoclopramide kinetics in plasma, respectively. The pharmacologic dose of metoclopramide resulted in a ∼2.2-fold increase in [^11^C]metoclopramide AUC_plasma_, while P-gp inhibition did not. AUC_liver_ was lower using the pharmacologic dose (42.9 ± 13.8 SUV·min) compared with the tracer dose (210.0 ± 32.4 SUV·min). P-gp inhibition enhanced the liver exposure in the pharmacologic condition only (81.0 ± 3.1 SUV·min). [^11^C]metoclopramide PET imaging suggests an unpredicted role for hepatocyte uptake transporter(s) in controlling metoclopramide pharmacokinetics in addition to the known contribution of the metabolic enzymes and the P-gp.

## 1. Introduction

Recent pharmacokinetic studies have clearly established that the liver metabolism of drugs is not solely governed by the activity of metabolizing enzymes. The hepatobiliary clearance of many drugs is controlled by the interplay of metabolizing enzymes and membrane transporters at the hepatocyte level [1]. Several uptake transporters expressed at the sinusoidal membrane of hepatocytes mediate the cellular import of many drugs and their subsequent exposure to metabolizing enzymes in hepatocytes [2]. Then, drugs and/or newly produced metabolites can be excreted to the bile by efflux transporters, mainly ATP-binding cassette (ABC) transporters, expressed at the canalicular membrane, including the P-glycoprotein (P-gp, ABCB1) [3].

Metoclopramide is a widely prescribed antiemetic drug for the treatment of esophageal reflux, dyspepsia, gastroparesis, and chemotherapy-related nausea [4]. It has been shown that metoclopramide is mainly eliminated through hepatic metabolism [5]. Hepatic impairment such as liver cirrhosis was associated with a 50% lower plasma clearance and a greater half-life [6], and only 19% of metoclopramide is excreted unchanged in urine after intravenous administration [7]. The parameters that govern the liver metabolism of metoclopramide remain poorly documented. A nonlinear bioavailability of metoclopramide has been reported in rats, suggesting a role for saturable systems [8]. Metoclopramide is a known substrate of the human and rodent P-gp [9, 10]. In mice, P-gp deficiency was shown to enhance the brain distribution of metoclopramide, with no or negligible impact on its plasma pharmacokinetics (PK) [9, 10]. To the best of our knowledge, the importance of carrier-mediated systems at the liver level on metoclopramide PK has not yet been investigated.

It is nonetheless difficult to link the intrinsic role of membrane transporters and metabolizing enzymes to the production of drug metabolites using conventional PK, that is, drug determination in plasma [11]. To that end, positron emission tomography (PET) imaging using radiolabeled analogues of drugs is an appealing approach to study the tissue distribution in a noninvasive manner [12]. This strategy offers the opportunity to noninvasively quantify the impact of membrane transporters on drug distribution in clearance and nonclearance organs [12, 13]. Metoclopramide displays a methoxy moiety on the aromatic core, making this structure an excellent candidate for isotopic labeling with carbon-11. This strategy offers the great advantage of being able to label the drug without modifying its structure and biological properties. We recently developed [^11^C]metoclopramide as a PET probe to study the impact of P-gp function on the blood-brain barrier (BBB) [14].

The present study aimed to elucidate the impact of liver uptake transport on the plasma kinetics, metabolism, and liver accumulation of metoclopramide in vivo. To that end, we report the isotopic labeling of metoclopramide with carbon-11 starting from the corresponding *O*-desmethyl precursor synthesized in one step from metoclopramide itself. The liver kinetics of [^11^C]metoclopramide were studied using PET imaging in rats to reveal the importance of carrier-mediated systems on metoclopramide PK.

## 2. Materials and Methods

### 2.1. Chemicals

Chemicals including metoclopramide hydrochloride were purchased from Aldrich (France) and used as received. Tariquidar used for P-gp inhibition was purchased from Eras Labo (France). Tariquidar solutions for i.v. injection (4.4 mg·mL^−1^) were prepared on the day of experiment by dissolving tariquidar dimesylate 2.35 H_2_O (∼6 mg) in a 5% (*w* : *v*) dextrose solution (0.5 mL) followed by dilution with sterile water (0.5 mL). Metoclopramide was administered using sterile Metoclopramide Renaudin® for i.v. injection (10 mg/2 mL, France).

### 2.2. Chemistry

Reactions were monitored by thin-layer chromatography (TLC) on aluminum precoated plates of silica gel 60F_254_ (VWR, France). Flash chromatography was conducted on a silica gel (0.63–0.200 mm, VWR, France) column. The compounds were localized at 254 nm using a UV-lamp. ^1^H NMR and ^13^C NMR spectra were recorded on a Bruker Advance 400 MHz apparatus using CDCl_3_ as a solvent. The chemical shifts (*δ*) are reported in ppm, downfield from TMS (*s*, *t*, *m*, and *b* for singlet, triplet, multiplet, and broad signals, resp.), and referenced with the solvent residual chemical shift. High-resolution mass spectrometry (HRMS) analysis was performed by the small molecule mass spectrometry platform of IMAGIF (Gif-sur-Yvette, France, http://www.imagif.cnrs.fr), by electrospray with positive (ESI+) ionization mode.

4-Amino-5-chloro-*N*-(2-(diethylamino) ethyl)-2-hydroxybenzamide (**1**) was prepared from metoclopramide hydrochloride. Metoclopramide hydrochloride (0.5 g, 1.49 mmol) was dissolved in aqueous hydrobromic acid (48%, 3 mL), and the solution was refluxed for 2 h. Upon cooling to 0°C, aqueous ammonia solution (25%, 10 mL) was added to reach a pH of 9. The aqueous solution was extracted with ethyl acetate (3 × 5 mL), and the combined organic layers were washed with brine (5 mL), dried over sodium sulfate, and filtered, and then the solvent was evaporated. The crude product was purified by flash chromatography on a short pad of silica gel (ethyl acetate/methanol, 9 : 1 *v*/*v*) to afford compound **1** (300 mg, 71%) as a colorless oil. ^1^H NMR (400 MHz, CDCl_3_): *δ* 7.30 (s, 1H, CH_Ar_), 7.15 (b, 1H, NH), 6.26 (s, 1H, CH_Ar_), 4.41 (b, 2H, NH_2_), 3.42 (t, *J* = 5.2 Hz, 2H, CH_2_), 2.55–2.65 (m, 6H, 3 × CH_2_), and 1.03 (t, *J* = 7.2 Hz, 6H, 2 × CH_3_) ppm. ^13^C NMR (101 MHz, CDCl_3_): *δ* 169.1 (C=O), 161.7 (C^IV^), 147.7 (C^IV^), 126.6 (CH), 109.4 (C^IV^), 106.0 (C^IV^), 102.4 (CH), 51.2 (CH_2_), 46.6 (2 × CH_2_), 36.6 (CH_2_), and 11.6 (2 × CH_3_) ppm. HR-ESI(+)-MS confirmed the absence of metoclopramide in the final preparation. The calculated *m*/*z* value for C_13_H_21_ClN_3_O_2_ is 286.1244 [M + H]+. The found value was 286.1248 for the preparation.

### 2.3. Radiochemistry

Automated radiosynthesis of [^11^C]metoclopramide from **1** was performed using a TRACERlab FX C Pro synthesizer (GE Healthcare, USA) (available here). No carrier-added [^11^C]CO_2_ (60–80 GBq) was produced via the ^14^N(*p*, *α*)^11^C nuclear reaction by irradiation of a [^14^N]N_2_ target containing 0.15–0.5% of O_2_ on a Cyclone 18/9 cyclotron (18 MeV, IBA, Belgium). [^11^C]CO_2_ was subsequently reduced to [^11^C]CH_4_ and iodinated to [^11^C]CH_3_I following the process described by Larsen et al. [15] and finally converted to [^11^C]CH_3_OTf according to the method of Jewett [16]. [^11^C]CH_3_OTf was bubbled into a solution of *O*-desmethyl-metoclopramide **1** (1 mg) and aqueous sodium hydroxide (3 M, 7 *μ*L) in acetone (400 *μ*L) at −20°C for 3 min. The mixture was heated at 110°C for 2 min followed by evaporation of the residual solvent to dryness at 110°C under vacuum for 30 s. Upon cooling down to 60°C, a mixture of aqueous NaH_2_PO_4_ (20 mM)/acetonitrile/H_3_PO_4_ (85/15/0.2 *v*/*v*/*v*) was added. Purification was realized by reversed-phase HPLC (Waters Symmetry® C18 7.8 × 300 mm, 7 *μ*m) with a 501 HPLC pump (Waters, USA) using aqueous NaH_2_PO_4_ (20 mM)/acetonitrile/H_3_PO_4_ (85/15/0.2 *v*/*v*/*v*, 5 mL/min) as an eluent. UV detection (K2501, Knauer, Germany) was performed at 220 nm. The purified compound was diluted with water (20 mL) and passed through a Sep-Pak® C18 cartridge (Waters, USA). The cartridge was rinsed with water (10 mL) and eluted with ethanol (2 mL), and the final compound was diluted with saline (0.9% *w*/*v*, 8 mL) to afford ready-to-inject [^11^C]metoclopramide.

### 2.4. Quality Control

Quality control was performed on three consecutive runs using a 717_plus_ Autosampler HPLC system equipped with a 1525 binary pump and a 2996 photodiode array detector (Waters, USA) and a FlowStar LB 513 gamma detector (Berthold, France). The system was monitored with the Empower 3 (Waters) software. HPLC was realized on a reversed-phase analytical Symmetry C18 (150 × 3.9 mm, 5 *μ*m, Waters) column using a mixture of aqueous NaH_2_PO_4_ (4 mM)/acetonitrile/H_3_PO_4_ (90/10/0.2 *v*/*v*/*v*, 2 mL/min) as an eluent. UV detection was performed at 274 nm. Identification of the peak was assessed by comparing the retention time of [^11^C]metoclopramide with the retention time of the nonradioactive metoclopramide reference (*t*_R_^ref^). For acceptance, the retention time must be within the *t*_R_^ref^ ± 10% range. Radiochemical and chemical purities were calculated as the ratio of the area under the curve (AUC) of the compound peak to the sum of the AUCs of all other peaks on gamma and UV chromatograms, respectively. Radiochemical and chemical purities are the mean values of three consecutive runs. Molar activity was calculated as the ratio of the activity of the collected peak of [^11^C]metoclopramide measured with an activimeter (Capintec®, Berthold) to the molar quantity of metoclopramide determined using calibration curves derived from the UV chromatogram. Molar activity is calculated as the mean value of three consecutive runs. Shelf stability was measured one hour after the end of the synthesis with a repeated run of quality control.

### 2.5. Animals

[^11^C]metoclopramide PET kinetics in the liver were measured in 18 male Wistar rats (mean weight = 322 ± 23 g) (Janvier, France). The study was conducted in accordance with the French legislation and European directives on the use of animals in research. The protocol has been accepted by a local ethics committee for animal use (protocol AFAPIS A16/057).

### 2.6. PET Imaging

PET scans were performed using an Inveon® microPET system (Siemens, Germany) at room temperature. Anesthesia was induced and thereafter maintained using 3% and 1.5–2.5% isoflurane in O_2_, respectively. A transmission scan with a rotating cobalt-57 source, for attenuation correction, was performed. Thirty-minute dynamic acquisitions were performed, starting from i.v. injection of a bolus [^11^C]metoclopramide in a catheter inserted in the caudal lateral vein. Four different conditions were tested: a tracer dose of [^11^C]metoclopramide (39.4 ± 9.0 MBq; 4.3 ± 3.9 *µ*g/kg; 14.5 ± 13.2 nmol/kg) was injected either in the absence (*n*=5) or in the presence (*n*=4) of the P-gp inhibition. Coinjection of [^11^C]metoclopramide with unlabeled metoclopramide (36.7 ± 7.9 MBq; 3 mg/kg; 10 *µ*mol/kg) was also performed in the absence (*n*=4) and the presence (*n*=5) of P-gp inhibition. To that end, unlabeled metoclopramide (∼0.2 mL) was added to the [^11^C]metoclopramide solution for a total injected volume <0.8 mL. P-gp inhibition was obtained using tariquidar (8 mg/kg, i.v. bolus <0.65 mL) administered 15 min before PET.

Dynamic PET images were reconstructed using the FORE+OSEM2D algorithm including normalization, attenuation, and scatter and random corrections. Image analysis and quantification of radioactivity uptake were performed using PMOD® software (version 3.5, PMOD Technologies LLC, Switzerland). A 5 mm diameter sphere was drawn on the upper part of the median lobe of the liver to generate the corresponding time-activity curves (TACs) with a time frame duration of 0.25 min; 0.5 min × 2; 0.75 min; 1 min × 4; 1.5 min; 2 min × 4; and 2.5 and 3 min × 4. Radioactivity was corrected for carbon-11 decay, injected dose, and animal weight to express the measurements in standardized uptake value (SUV).

### 2.7. Data Analysis

Liver exposure to [^11^C]metoclopramide was estimated in all conditions using the mean area under the curve of the tissue radioactivity from 0 to 30 min (AUC; SUV·min).

Imaging data were interpreted in the light of [^11^C]metoclopramide plasma kinetics measured from 0 to 60 min, previously reported in the same experimental conditions (*n*=3 animals per condition) [14]. [^11^C]metoclopramide AUC in plasma from 0 to 60 min was calculated in all conditions to assess the respective impact of pharmacologic dose and P-gp inhibition on [^11^C]metoclopramide plasma exposure. Plasma AUC was corrected for injected radioactivity and animal weight (SUV·min units) and is therefore inversely correlated to [^11^C]metoclopramide plasma clearance (plasma clearance = dose/AUC_plasma_).

The mean plasma kinetics of parent [^11^C]metoclopramide obtained in each respective condition (4 conditions, *n*=3 per condition) [14] were fitted using a one-phase exponential decay function. The fitted curve was used as the arterial plasma input function to describe the transfer of [^11^C]metoclopramide from plasma to the liver. The transfer constant (*k*_uptake_) was calculated as previously described using an integration plot method [17]. The following equation was used:

(1) $$\begin{array}{c} \frac{X_{t,\text{liver}}}{C_{t,\text{blood}}}=k_{\text{uptake}}\;\times \;\frac{{\text{AUC}}_{0-t,\text{blood}}}{C_{t,\text{blood}}}+V_{E}, \end{array}$$

where *X*_*t*,liver_ is the amount of radioactivity per gram tissue in the liver at time *t* and *C*_*t*, blood_ is the radioactivity concentration in plasma at time *t*. AUC_0−*t*,blood_ represents the AUC of parent [^11^C]metoclopramide in plasma from 2 min to time *t*. The *k*_uptake_ can be obtained by performing linear regression analysis of a plot of *X*_*t*,organ_/*C*_*t*,blood_ versus AUC_0−*t*,blood_/*C*_*t*,blood_ and calculating the slope of the regression line which was linear from 2 to 7.5 min in all tested conditions. The unit of *k*_uptake_ is mL of blood per min per gram tissue (mL/min/g of tissue). *V*_*E*_ is the *y*-intercept of the integration plot.

### 2.8. Statistical Analysis

Statistical analysis was performed using the GraphPad Prism 7.0 software (GraphPad Inc., CA, USA). Variance homogeneity was assessed using Levene's test (*p* > 0.05). Data were compared using a one-way ANOVA followed by a Tukey post hoc test. A result was deemed significant when a two-tailed *p* value was less than 0.05.

## 3. Results

### 3.1. Chemistry

**Figure 1:**
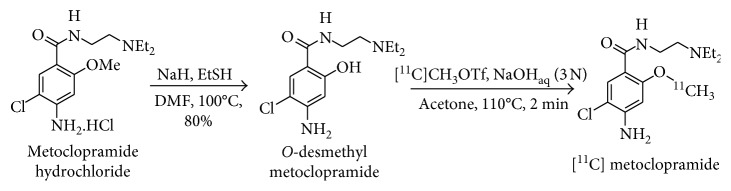
Synthesis of the desmethyl precursor from metoclopramide hydrochloride and radiosynthesis of [^11^C]metoclopramide.

Metoclopramide can be isotopically labeled with carbon-11 at the 2-methoxy moiety of the aromatic ring. For that purpose, commercially available metoclopramide was demethylated in one step in the presence of refluxing hydrobromic acid to afford the *O*-desmethyl-metoclopramide precursor **1** in 71% yield within 2 hours (Figure 1). A treatment with an aqueous ammonia solution was realized to afford **1** as the free base to be used directly for the radiosynthesis of [^11^C]metoclopramide.

Different conditions were explored to carry out the radiomethylation, and aqueous sodium hydroxide was revealed to be the most appropriate base for the reaction. Stronger bases such as sodium methanoate gave lower yields, whereas side products resulting from the deprotonation of the aniline or the amide moiety were observed when using sodium hydride. No significant influence of the solvent was noted when using either acetone or *N*,*N*-dimethylformamide (Figure 1). Finally, [^11^C]metoclopramide (1.4–2.0 GBq) was radiosynthesized in 10% radiochemical yield with a radiochemical purity above 98% and a molar activity of 130 ± 20 GBq/*μ*mol.

### 3.2. In Vivo Experiments

**Figure 2:**
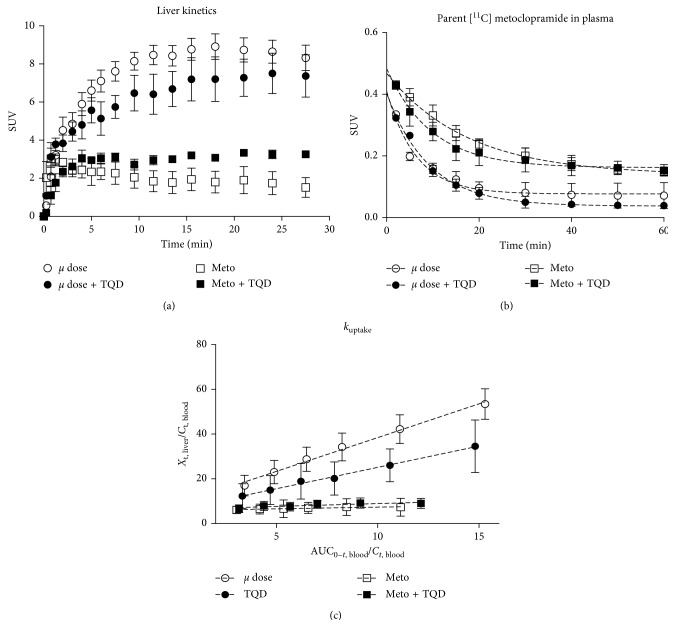
PET kinetics in the rat liver (*n*=4‐5) were measured in the absence (*µ* dose) and the presence of a pharmacologic dose of metoclopramide (3 mg/kg, Meto), with or without P-gp inhibition using i.v. tariquidar (8 mg/kg) (TQD). Time-activity curves of radioactivity obtained in the liver after injection of [^11^C]metoclopramide are shown in (a). The fitted curves (dashed lines) corresponding to the mean plasma kinetics of parent [^11^C]metoclopramide are shown in (b). Plasma kinetics were obtained from previously reported experiments performed in the same conditions (*n*=3 animals per condition) [14]. The mean integration plots (dashed line) used to calculate the transfer constant of [^11^C]metoclopramide from plasma to the liver are shown in (c). See Materials and Methods for definition of variables used in integration plot analysis. The liver uptake rate constants correspond to the slope of the linear regression lines, calculated from 2 to 7.5 min after injection. Data shown are mean ± SD.

**Figure 3:**
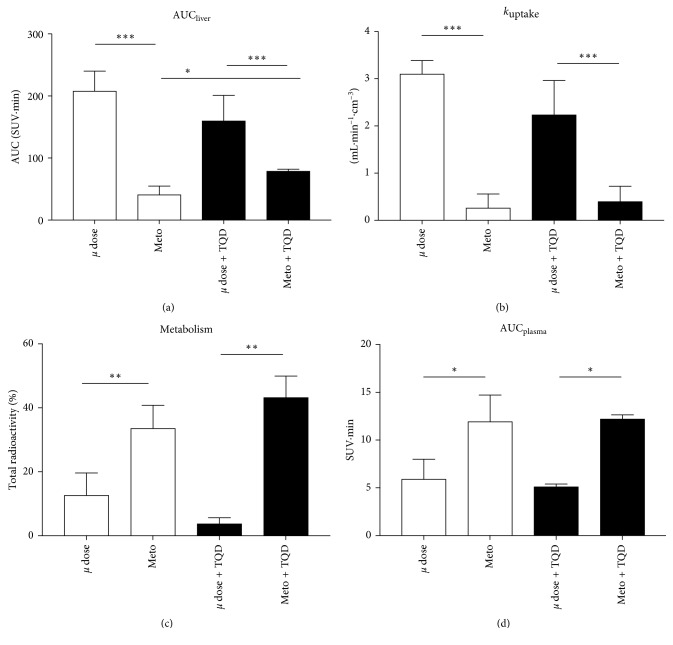
[^11^C]metoclopramide liver kinetics in rats (*n*=4‐5) were compared in the absence (*µ* dose) and the presence of a pharmacologic dose of metoclopramide (3 mg/kg, Meto), with or without P-gp inhibition using i.v. tariquidar (8 mg/kg) (TQD). Liver exposure to [^11^C]metoclopramide was described by the area under the curve (AUC) of the radioactivity kinetics in the liver (a). The rate constant of [^11^C]metoclopramide uptake by the liver was calculated using the integration plot analysis (b). The fraction of parent [^11^C]metoclopramide kinetics in plasma 60 min after injection is shown in (c). The plasma exposure to parent [^11^C]metoclopramide is expressed as AUC in (d). Data regarding plasma kinetics and metabolism were calculated from Pottier et al. [14]. Data shown are mean ± SD. Statistical significance is as follows: ^∗^ *p* < 0.05; ^∗∗^ *p* < 0.01; and ^∗∗∗^ *p* < 0.05.

In the liver, the pharmacologic dose of metoclopramide dramatically decreased the accumulation of radioactivity compared with the tracer dose (Figure 2). Liver AUC was significantly lower using the pharmacologic dose compared to the tracer dose in both the presence and the absence of P-gp inhibition (Figure 3).

In plasma, coinjection with a pharmacologic dose of metoclopramide resulted in higher plasma concentrations (SUV) compared with the tracer dose [14] with significant 2.1-fold and 2.3-fold increases in AUC_plasma_ in the absence and the presence of P-gp inhibition, respectively (Figure 3). The proportion of parent [^11^C]metoclopramide in plasma was higher using the pharmacologic dose of metoclopramide compared to the tracer dose. P-gp inhibition had nonsignificant impact on [^11^C]metoclopramide metabolism up to 60 min after injection (Figure 3).

The liver uptake of [^11^C]metoclopramide (*k*_uptake_) was significantly lower using a pharmacologic dose (Figures 2 and 3). P-gp inhibition did not impact [^11^C]metoclopramide *k*_uptake_ in either the tracer dose or the pharmacologic dose condition (Figures 2 and 3). In the tracer dose condition, P-gp inhibition did not significantly impact liver AUC. In the pharmacologic dose condition, P-gp inhibition resulted in an increased liver AUC (Figure 3).

## 4. Discussion

*O*-desmethyl metoclopramide **1** was synthesized in one step from commercially available metoclopramide in a straightforward approach. A one-step procedure has previously been proposed by Monkovic et al. using sodium ethanethiolate freshly prepared from ethanethiol and sodium hydride [18]. To circumvent the use of ethanethiol, demethylation of metoclopramide can be alternatively performed in one step in the presence of refluxing hydrobromic acid. This method afforded the *O*-desmethyl metoclopramide precursor **1** in 71% yield within 2 hours. In comparison with the approach proposed by Monkovic et al., the presented method affords the desired desmethyl compound in lower yield (71% and 98%, resp.) and comparable reaction times (2 h and 90 min, resp.) but avoids the preparation of ethanethiolate from malodorous and hazardous ethanethiol. A treatment with aqueous ammonia was performed to afford **1** as a free base. Precursor **1** could therefore be used directly for the radiosynthesis of ^11^[C]metoclopramide.

In this study, we studied the liver kinetics of [^11^C]metoclopramide in rats to elucidate the dose-dependent PK and metabolism observed in vivo [8, 14]. Our results suggest that a saturable transport by the liver, which did not correspond to P-gp function, is critical for metoclopramide liver clearance, metabolism, and PK.

First, the role of P-gp function in [^11^C]metoclopramide kinetics was investigated using tariquidar, a potent P-gp inhibitor. The importance of the interaction of tariquidar with hepatocytes of PK remains poorly investigated. *In vitro*, tariquidar is an inhibitor of OATP2B1 (SLCO2B1) expressed at the sinusoidal membrane of hepatocytes, but not OATP1B1 (SLCO1B1) or OATP1B3 (SLCO1B3) [19]. In humans, tariquidar was shown to inhibit P-gp at the canalicular membrane and to enhance the liver exposure to [^99m^Tc]sestamibi, a radiolabeled substrate probe [20]. In the pharmacologic condition, P-gp inhibition increased metoclopramide accumulation in the liver with no impact on the liver uptake. This differential effect may be explained by the specific role of P-gp in hepatocytes, which mediates the biliary efflux of many drugs [2]. The P-gp effect was not significant in the tracer dose situation probably because the radioactivity in the liver is mainly composed of radiometabolites which may not be transported by P-gp [14].

Interestingly, P-gp inhibition did not impact [^11^C]metoclopramide plasma clearance and metabolism over 60 min in the limited investigation time followed by carbon-11 decay. Metoclopramide PK in mice (3 mg/kg) has been reported and showed no influence of P-gp deficiency over 5 hours [10]. Thus, metoclopramide dose-dependent plasma clearance cannot be attributed to the saturation of P-gp secretion at the biliary level, which is consistent with the fact that metoclopramide is not a P-gp inhibitor [9]. P-gp-mediated biliary secretion may nonetheless control the liver accumulation with low or hardly detectable signature in measurable plasma PK.

Using a coinjection strategy, we showed that the liver uptake, rather than P-gp-mediated biliary secretion, is a major determinant of [^11^C]metoclopramide metabolism and plasma clearance *in vivo.* The uptake transport had major impact on liver exposure to metoclopramide and explains the dramatic differences in [^11^C]metoclopramide plasma clearance observed between the tracer dose and the pharmacologic dose condition. This suggests that a carrier-mediated uptake transport at the sinusoidal liver plasma membrane may control metoclopramide metabolism independent of the intrinsic activity of metabolizing enzymes.

The parameters that control metoclopramide pharmacokinetics (PK) in patients remain poorly documented. Metoclopramide is a substrate of the highly polymorphic cytochrome P450 2D6 (CYP2D6) which may account for patient-to-patient PK variability [21, 22]. In rats, a saturable first-pass metabolism, the mechanism of which is unknown, has been reported [8]. Little is known regarding the importance of interaction of metoclopramide with relevant hepatocyte transporters [23]. The literature reports that metoclopramide is not a substrate of the breast cancer resistance protein (ABCG2) [14], which is expressed with P-gp at the canalicular side of hepatocytes [2, 23]. Interestingly, *in vitro* studies have shown that metoclopramide is a substrate of human organic cation transporter 1 (OCT1, SLC22A1), a major uptake transporter expressed at the sinusoidal side of hepatocytes [24]. PET imaging using [^11^C]metoclopramide will be useful to assess the specificity and the relative importance of OCT1 and other hepatocyte transporters on metoclopramide liver uptake and PK [25].

Species differences in the hepatobiliary function and the carrier-mediated transport by hepatocytes have been reported between rats and humans [26]. This is a limitation for the clinical interpretation of our results. [^11^C]metoclopramide is the isotopic analogue of metoclopramide for which formulations for i.v. injection exist. [^11^C]metoclopramide PET imaging can thus be safely and noninvasively performed in humans using both the tracer and the pharmacological doses. Such a method was recently proposed to reveal the saturable uptake transport of erlotinib by the liver in healthy volunteers [27]. A similar approach could be performed in humans to address the role of hepatocyte transporter(s) in the PK variability of metoclopramide [4].

## 5. Conclusions

Using [^11^C]metoclopramide PET imaging in rats, we showed that the in vivo metabolism and plasma clearance of metoclopramide may depend on a saturable uptake transport by the liver. P-gp function is not a rate-limiting step for metoclopramide plasma clearance or metabolism but may nonetheless control metoclopramide exposure to the liver in the absence of detectable impact on plasma kinetics.

## Data Availability

Raw data are available from the corresponding author upon request.

## Supplementary Materials

Supplementary Materials Figure S1: Scheme of the TRACERlab FX C Pro synthesizer for the automated radiosynthesis of ^11^C-metoclopramide. (1) Reactor containing **1** (1 mg) and aqueous sodium hydroxide (3 M, 7 *μ*L) in acetone (400 *μ*L); (2) 0.5 mL of NaH_2_PO_4_ (20 mM)/CH_3_CN/H_3_PO_4_ (0.5 mL, 85/15/0.2 v/v/v); (3) reverse phase HPLC column (Waters Symmetry® C18 7.8 × 300 mm, 7 m); (4) 20 mL of water; (5) 10 mL of water; (6) 2 mL of ethanol; (7) 18 mL of aqueous 0.9% NaCl; and (8) Waters Sep-Pak® C18 cartridge. Figure S2: Semipreparative HPLC with (A) UV detection and (B) gamma detection for purification of ^11^C-metoclopramide.
