# Supplementary material for: Positron Emission Tomography Imaging Reveals an Importance of Saturable Liver Uptake Transport for the Pharmacokinetics of Metoclopramide
Source: Contrast Media Mol Imaging. 2018 May 8;2018:7310146. doi: 10.1155/2018/7310146 (PMC5964550; doi:10.1155/2018/7310146)
Supplement: Supplementary Materials — Figure S1: Scheme of the TRACERlab FX C Pro synthesizer for the automated radiosynthesis of 11C-metoclopramide. (1) Reactor containing 1 (1 mg) and aqueous sodium hydroxide (3 M, 7 μL) in acetone (400 μL); (2) 0.5 mL of NaH2PO4 (20 mM)/CH3CN/H3PO4 (0.5 mL, 85/15/0.2 v/v/v); (3) reverse phase HPLC column (Waters Symmetry® C18 7.8 × 300 mm, 7 m); (4) 20 mL of water; (5) 10 mL of water; (6) 2 mL of ethanol; (7) 18 mL of aqueous 0.9% NaCl; and (8) Waters Sep-Pak® C18 cartridge. Figure S2: Semipreparative HPLC with (A) UV detection and (B) gamma detection for purification of 11C-metoclopramide. [file 7310146.f1.docx]

**Positron Emission Tomography Imaging Reveals an Importance of Saturable Liver Uptake Transport for the Pharmacokinetics of Metoclopramide**

Fabien Caillé^1^, Sébastien Goutal^1^, Solène Marie^1^, Sylvain Auvity^1,2^, Salvatore^2,3^ Cisternino^2,3^, Bertrand Kuhnast^1^, Géraldine Pottier^1^, Nicolas Tournier^1,*^

1. Imagerie Moléculaire In Vivo, IMIV, CEA, Inserm, CNRS, Univ. Paris-Sud, Université Paris Saclay, CEA-SHFJ, Orsay, F-91400, France
2. Variabilité de réponse aux psychotropes, UMR-S 1144, Inserm, Université Paris Descartes, Université Paris Diderot, Paris, F-75006, France
3. Assistance Publique des Hôpitaux de Paris – AP-HP, Paris, France

**^*^Corresponding author:** Nicolas TOURNIER

nicolas.tournier@cea.fr

CEA, DSV, Joliot, Service Hospitalier Frédéric Joliot, Orsay, F-91401, France

Phone number: +33 1 69 86 77 12 Fax number: +33 1 69 86 77 68


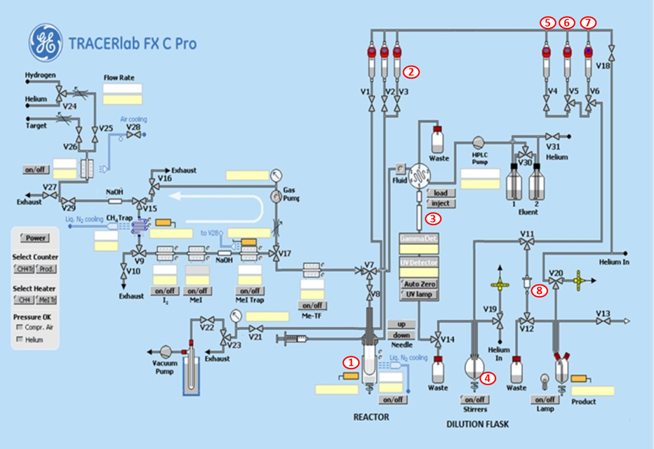


**Figure S1.** Scheme of the TRACERlab FX C Pro synthesizer for the automated radiosynthesis of ^11^C-metoclopramide. (1) Reactor containing **1** (1 mg) and aqueous sodium hydroxide (3 M, 7 μL) in acetone (400 μL); (2) 0.5 mL of NaH_2_PO_4_ (20 mM) / CH_3_CN / H_3_PO_4_ (0.5 mL, 85/15/0.2 v/v/v); (3) reverse phase HPLC column (Waters Symmetry^®^ C18 7.8 x 300 mm, 7 μm); (4) 20 mL of water; (5) 10 mL of water; (6) 2 mL of ethanol; (7) 18 mL of aqueous 0.9% NaCl; (8) Waters Sep-Pak^®^ C18 cartridge


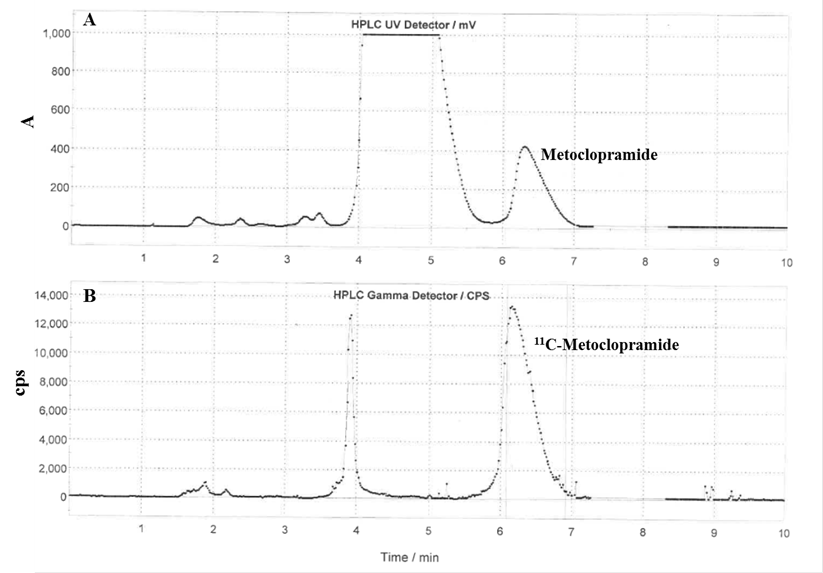


**Figure S2.** Semi-preparative HPLC with (A) UV detection and (B) gamma detection for purification of ^11^C-metoclopramide.
